# Evaluating the Effectiveness of Earplugs in Preventing Noise-Induced Hearing Loss in an Auto Parts Factory in China

**DOI:** 10.3390/ijerph18137190

**Published:** 2021-07-05

**Authors:** Wei Gong, Liangliang Zhao, Ling Li, Thais C. Morata, Wei Qiu, Huiling Amy Feng, Baoli Zhu

**Affiliations:** 1 Jiangsu Provincial Center for Disease Control and Prevention, Nanjing 210009, China or wrm3@cdc.gov (W.G.); zhaoll@jscdc.cn (L.Z.); liling0331@jscdc.cn (L.L.); 2 National Institute for Occupational Safety and Health (NIOSH), Centers for Disease Control and Prevention (CDC), Cincinnati, OH 45226, USA; TMorata@cdc.gov (T.C.M.); haf0@cdc.gov (H.A.F.); 3 Auditory Research Laboratories, State University of New York at Plattsburgh, Plattsburgh, NY 12903, USA; Qiuw@plattsburgh.edu; 4 Center for Global Health, School of Public Health, Nanjing Medical University, Nanjing 211166, China

**Keywords:** hearing protection device (HPD), earplug, noise-induced hearing loss (NIHL), fit testing, personal attenuation rating (PAR), audiometric test

## Abstract

A survey was administered to 385 noise-exposed workers from an auto parts factory and 1268 non-noise-exposed health department employees in China. Individual 8 h A-weighted equivalent sound levels (L_Aeq,8h_), earplug personal attenuation ratings (PARs), and pure-tone audiometric tests were performed. The average L_Aeq,8h_ of noise-exposed workers was 87 dB (A) with a mean PAR of 7 dB. The prevalence of high-frequency hearing loss was 65% for noise-exposed workers and 33% for the non-noise-exposed employees. The use of earplugs had no observable effect on the prevalence of high-frequency hearing loss of the study participants (OR 0.964, 95% CI 0.925–1.005, *p* = 0.085). No significant relationship between the effectiveness offered by earplug use and high-frequency hearing thresholds at 3, 4, and 6 kHz was found (t = −1.54, *p* = 0.125). The mandatory requirement of earplug use without individualized training on how to wear HPDs correctly had no detectable effect on the prevention of hearing loss at the auto parts factory. The hearing conservation program at the surveyed factory was not effective. Periodic hearing tests, earplug fit testing, expanding the offer of different types of hearing protection, and employee education about the importance of protecting their hearing were recommended to the occupational health and safety program.

## 1. Introduction

Noise-induced hearing loss (NIHL) is the third most prevalent chronic physical condition among adults in the United States [1]. In China, there were 1220 cases of occupational noise-induced deafness in 2016 with an annual growth rate of 24.2% from 2014 reported in the national occupational health surveillance system [2]. In the field of occupational health, a hierarchy of controls that prioritizes controlling the source of exposure is recommended for being more effective, while less burdensome to workers and less dependent on behavior than those involving personal protective equipment. However, in practice (and from the literature) the provision of hearing protection devices (HPDs) is the most common approach to reduce noise exposure [3,4]. A large epidemiologic evaluation, which examined the association between self-reported hearing protection use at work and hearing thresholds of 19,911 noise-exposed U.S. workers, observed that hearing threshold differences of workers who reported “never” versus “always” wearing hearing protection was marginally significant. A significant linear trend towards increased risk of high-frequency threshold shift with decreased use of hearing protection was also observed [5]. The evidence of the effectiveness of HPDs seems dependent on several factors such as inconsistencies in earplug use, lack of HPD alternatives, the skill of the worker to fit a device, and the instructions for insertion [4].

In China, workers are required to wear HPDs when they are exposed to workplace noise levels above 85 dB (A) over 8 h [6]. The single number rating (SNR) found on the product’s label indicates a level of attenuation that the device can offer. However, studies have repeatedly shown that these possible levels of noise attenuation are rarely achieved in field conditions [4,7,8]. A median personal attenuation rating (PAR) of 10 dB for foam earplugs was reported in a recent study at four factories in China, well below the labeled SNR of 25 dB [9]. Some workers reported they did not wear HPDs all the time during their work shifts. Another recent study showed that the proportion of regular HPD use increased as the noise exposure level increased [10]. The reports examined the effectiveness of the earplugs by using self-reported data or questionnaire information but did not provide PARs obtained from noise-exposed workers [5,10].

Since HPDs are widely used in noisy industrial environments, it is essential to determine whether HPDs are effective in preventing noise-induced hearing loss. This study at an auto parts factory in China was designed to investigate the attenuation offered by earplugs and their effectiveness in preventing NIHL.

## 2. Materials and Methods

This study was approved by the Ethics Committee of Jiangsu Provincial Center for Disease Control and Prevention (JSCDC). All participants provided their consent to participate in the study. Information obtained by a questionnaire and company data were used to obtain historical noise exposure information.

### 2.1. Selection of Participants and Background Information about the Surveyed Factory

This case-control study was conducted at an auto parts factory and the Jiangsu Provincial health department in eastern China. JSCDC conducted an initial investigation for participants routinely exposed to occupational noise at an auto parts factory in August 2018. A second investigation for participants who were not exposed to occupational noise was conducted from December 2018 to September 2019. The non-noise-exposed workers were employees from the Jiangsu Provincial health department, including two clinical hospitals and 10 local centers for Disease Control and Prevention. Each participant was given an otoscopic exam, tympanometry, and pure-tone audiometry (500–8000 Hz). The inclusion criteria were (1) no history of prior employment in a high noise environment, (2) no military service history or shooting activities, (3) a clear view of the tympanic membrane on otoscopic examination, and (4) type A tympanogram. To be eligible for participation, noise-exposed workers had to have a minimum of one year of employment at their current job. A total of 385 noise-exposed workers and 1268 non-noise-exposed employees met the eligibility criteria from original pools of 504 and 1672, respectively.

The production area of the surveyed auto parts factory consisted of two separated one-story buildings. Each building had multiple production lines and eight job titles (e.g., cutting, grinding, numerical control machine, heat treatment, sorting, packing, forklift driver, and inspection). Workers were not exposed to ototoxicants at this factory.

There were no physical barriers/walls between lines. The factory operated three rotating 8 h shifts, 5 days a week. Overtime work was available but not mandatory. The auto parts factory had a full-time health and safety manager who provided safety and health training and organized occupational health hazard monitoring and health surveillance. Part-time safety supervisors in each production area on each work shift assisted the safety manager. They were responsible for the field safety and occupational issues of the specific production area.

Noise-exposed workers were offered earplugs for voluntary use when the company started operations in 2003. In 2012, the use of 3M 1270 premolded earplugs became mandatory during workers’ full work shifts. It was the only type and model of earplug provided to the workers since then. The labeled attenuation value (SNR) for this earplug is 25 dB. All workers received pre-employment training and annual refresher training thereafter. The pre-employment training included environmental, safety, and health knowledge. The annual refresher consisted of group presentations including how to wear earplugs correctly with a written examination afterward.

### 2.2. Questionnaire Survey

Each participant was required to complete a health questionnaire, which was administered through a face-to-face interview. The following information was collected: demographic information (age, gender, etc.), noise exposure history (factory; work title; length of employment; duration of daily noise exposure; personal life habits such as shooting, smoking, and drinking), and history of ear diseases and use of ototoxic drugs. Noise-exposed employees were required to provide their HPD use history.

### 2.3. Personal Noise Exposure Levels

Area full-shift noise measurements were conducted at the worksite of the participants who were not exposed to noise using dBadge noise dosimeters (Casella, Sterling, MA, USA). Each dosimeter was calibrated before and after the measurement using a Casella CEL-120/2 Sound Level Calibrator. The noise measurement protocol was consistent with NIOSH recommendations for workplace noise exposure sampling [11]. The equivalent continuous A-weighted sound pressure level over 8 h (L_Aeq,8h_) data were extracted from the dosimeters directly, regardless of the length of the shift.

Each noise-exposed worker from the auto parts factory wore an ASV5910-R digital recorder (Hangzhou Aihua Instruments Co., Hangzhou, China), operating continuously with 16-bit resolution at a 48 kHz sampling rate, during their 8 h shifts. This instrument was selected because of our interest in the temporal characteristics of the noise exposure. The recorders comply with IEC 61672 and IEC 61252 standards and were used in previous studies [12,13]. The recorders were equipped with a ¼-inch microphone attached to workers’ shoulders below their ear and out of range of head movement. Before the measurement, each dosimeter was calibrated using an AWA6221 Sound Level Calibrator (Aihua Instruments Co., Hangzhou, China). Immediately after recordings were completed, the data were transferred from the recorder to a computer for subsequent L_Aeq,8h_ and kurtosis calculation. To address the non-Gaussian noise in this study, the kurtosis of the recorded noise signal was computed for consecutive 40 s time windows without overlap over the full shift using MATLAB software [14]. The mean kurtosis of these 40 s windows was calculated and used as the kurtosis value for the entire shift. The kurtosis value of the steady-state (Gaussian) noise is 3. Sounds having greater excursion, or higher peak amplitudes relative to baseline amplitudes, have greater kurtosis [14].

The L_Aeq,8h_ for noise-exposed workers was calculated based on the individual full-shift noise recording data using a program written with MATLAB (MathWorks, R2017) software. This protocol was consistent with NIOSH recommendations for workplace noise exposure sampling [11]. Because workers were required to wear HPDs after 2012, their adjusted L_Aeq,8h_ (AL_Aeq,8h_) is

AL_Aeq,8h_ = L_Aeq,8h_ − PAR (1)

The cumulative noise exposure (CNE), a composite noise exposure index based on L_Aeq_,_8h_ and service years, was used to quantify the cumulative noise exposure for each participant [15]. The CNE is defined as

CNE = L_Aeq,8h_ + 10 log T (2)

where T is the total worker service years.

Considering that this company only started its hearing protection program in 2012, the CNE for workers who started working before 2012 needed to be adjusted. The adjusted CNE (ACNE) was defined as

(3) $$\mathrm{ACNE}=10\log\left[T_{1}\times 10^{\frac{L_{\mathrm{Aeq},8h}}{10}}+T_{2}\times 10^{\frac{{\mathrm{AL}}_{\mathrm{Aeq},8h}}{10}}\right]$$

where T_1_ is the years of service before 2012 and T_2_ is the years of service after 2012. For example, if a worker had been working 9 years, the ACNE was calculated by L_Aeq,8h_ with T1 for the first 3 years and the AL_Aeq,8h_ with T_2_ for the last 6 years (keeping in mind that the investigation was conducted in 2018). If a worker started the job after 2012, then T_1_ = 0, and the ACNE was calculated by the AL_Aeq,8h_ for his work; T_2_ was the number of work years.

### 2.4. Fit Testing

The field attenuation estimation system used in this study was the 3M E-A-Rfit Dual-Ear Validation System [9], which incorporates an objective measurement method with field microphone-in-real-ear technology. The system consists of software and hardware, including a speaker with a digital sound processor, a dual-element microphone array, and surrogate probed test earplugs.

Fit testing was performed in a conference room at the local hospital. In 2018, 6 years after earplug use became mandatory, workers’ PARs were measured by asking the workers to fit the surrogate probed test earplugs by themselves the way they normally wore them during their work shift. No coaching or demonstration was provided. The PAR passing criterion was defined as being greater than the target minimum attenuation, which was based on the individual noise exposure results (see Section 2.3). This was set as the difference between the noise exposure and the exposure limit, which in China is 85 dB (A) (L_Aeq,8h_).

### 2.5. Audiometric Testing

All participants underwent pure-tone audiometry testing. Audiometry was performed in a double-walled, sound-treated booth at a local hospital or in a single-walled audiometric booth in the JSCDC mobile vehicle. A certified audiologist conducted the audiometric testing using a calibrated audiometer (Itera, GN Otometrics, Taastrup, Denmark). Air-conduction pure-tone hearing threshold (HT) levels were tested from 500 to 8000 Hz in each ear. For noise-exposed workers, hearing testing was conducted at least 16 h after the workers’ last occupational noise exposure. High-frequency hearing thresholds (HFHTs) used in the analysis were defined as the average thresholds at 3, 4, and 6 kHz in the better ear. The better ear was used because this was the criterion for establishing median hearing threshold levels of the control group [16]. Audiograms were classified as high-frequency hearing loss when, in either ear, thresholds at either 3, 4, or 6 kHz were equal to or greater than 30 decibels hearing level (dB HL).

### 2.6. Data Analysis

Descriptive statistics of information from questionnaires, noise measurements, PARs, and the prevalence of high-frequency hearing loss of each age group were calculated using Microsoft Excel. Because SNR is intended to be subtracted from the C-weighted sound level, while PAR is subtracted from the A-weighted sound level, 2 dB C minus A corrected SNR [17] was applied to compare PARs in this study.

Statistical analyses were performed with SAS (Release 9.4, SAS Institute, Inc., Cary, NC, USA). A statistical significance level of *p* < 0.05 was adopted for all analyses. The correlation between PAR and L_Aeq,8h_ was examined with the Pearson correlation coefficient. A one-way analysis of variance (ANOVA) was conducted to evaluate the difference of mean PAR by L_Aeq,8h_ group. To analyze the distribution characteristic of HTs at each frequency for all participants, a general linear model (GLM) was conducted, followed by Tukey’s studentized range (HSD) test.

Based on International Organization for Standardization (ISO)1999:2013 (using gender, age, L_Aeq,8h_, and service years), the estimated median high-frequency hearing threshold for each noise-exposed worker was calculated. The comparison of hearing thresholds between noise-exposed workers, non-noise-exposed employees, and ISO-estimated hearing threshold was conducted exclusively within gender by age group. The hearing threshold comparison was conducted by a GLM procedure following HSD test.

Two analyses were carried out to evaluate a possible association between earplug use and hearing outcome. First, correlations between the prevalence of high-frequency hearing loss and CNE, ACNE, earplug attenuation, age, and gender were examined by Pearson correlation analysis. Second, after confirming that smoking (F = 3.40, *p* = 0.066) and alcohol consumption (F = 0.28, *p* = 0.971) were not confounders using a generalized linear model, a final linear model “log (HFHT) = a + b (CNE) + c (CNE-ACNE) + age + gender” was conducted to check the association between HFHT with CNE, earplug protection, age, and gender. Log (HFHT) was used because the HFHT was not normally distributed.

## 3. Results

### 3.1. Participant Characteristics

A total of 385 noise-exposed workers and 1268 non-noise-exposed employees participated in this study. Among noise-exposed workers, 70% were male and 30% were female; their median duration of noise exposure was 8 years (25th–75th percentile: 4–11 years); their median age was 37 years with a standard deviation (SD) of 7 years. The male workers (35 ± 7.5 years) were younger than the female workers (40 ± 5.5 years) (F = 40.04, *p* < 0.001). For non-noise-exposed employees, 36% were male and 64% were female; their median tenure was 11 years (25th–75th percentile: 5–23 years); their mean age was 38 years with an SD of 11 years. The male employees (41 ± 10.6 years) were older than the female employees (37 ± 10.2 years) (F = 38.23, *p* < 0.001).

Walk-through observation during the investigation revealed that several workers did not wear earplugs all the time during their full shift even though they were required to do so. Some workers took the earplugs off when they needed to communicate.

### 3.2. Noise Exposure Levels

The area full-shift noise levels at non-noise-exposed employee workstations ranged from 65 to 78 dB (A), while the average L_Aeq,8h_ of noise-exposed workers in the auto parts factory was 87 dB (A). Noise exposures ranged from 77 to 102 dB (A) with an SD of 3.5 dB (A), with a mean kurtosis of 19.8 and an SD of 12.6.

**Figure 1:**
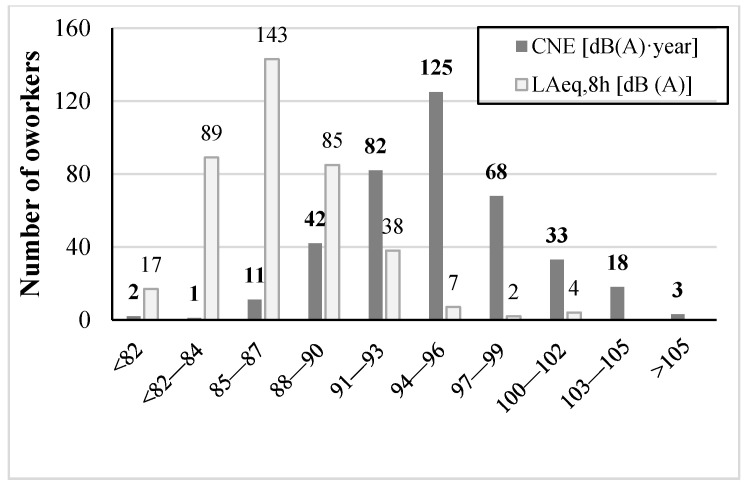
Noise exposure level (L_Aeq,8h_) and cumulative noise exposure (CNE) distribution of the noise-exposed workers. The mean L_Aeq,8h_ was 87 dB (A), ranging from 78 to 102 dB (A); CNE ranged from 80 to 111 dB (A)·year with a median level of 95 dB (A)·year.

The L_Aeq,8h_ estimates for 78.2% of the noise-exposed workers (*n* = 301) exceeded 85 dB (A). The highest level of noise was generated by the tube cutting, grinding, and machining processes. Figure 1 shows 37.1% (*n* = 143) of the workers had L_Aeq,8h_ between 85 and 87 dB (A), and 33.2% (*n* = 128) were exposed to levels that ranged from 88 to 93 dB (A). The median CNE level for all noise-exposed workers was 95 dB (A)·year, which ranged from 80 dB (A)·year to as much as 111 dB (A)·year.

### 3.3. Personal Attenuation Rating

**Figure 2:**
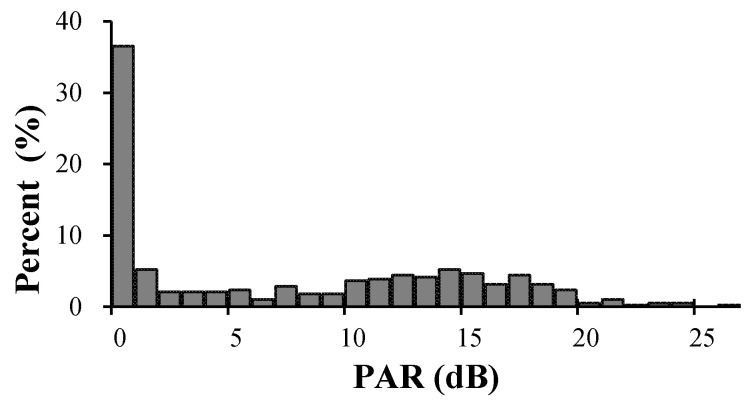
Histogram showing the distribution of personal attenuation ratings (PARs) achieved by percentage of the noise-exposed workers (*n* = 385) in this study.

Figure 2 reveals that PARs of noise-exposed workers ranged from 0 to 26 dB with a mean PAR of 7 dB and median PAR of 5 dB. Earplugs, as worn by the noise-exposed workers, did not provide any measurable noise attenuation for 37% (*n* = 149) of the workers. The SNR for this specific earplug is 25 dB. To compare these obtained PARs to the labeled attenuation values, a minus 2 dB correction was applied to the SNR [17]. Thus, the labeled attenuation rating of the tested earplug was adjusted to 23 dB. There were only five workers (1.3%) that achieve a PAR equal to or greater than 23 dB, while the actual attenuation value obtained by more than half of the workers was less than or equal to 5 dB.

In China, a 40% de-rating of the SNR is required in the workplace, which reduces the expected attenuation from this specific plug to 15 dB. The recorded mean PAR (i.e., 7 dB) was well below the SNR and de-rated SNR (15 dB). Twenty-one percent (*n* = 80) of the noise-exposed workers’ PARs achieved the de-rated SNR, while only one worker’s PAR achieved the SNR. The PAR passing rate was 53.7% among 315 workers whose noise exposure levels were 85 dB (A) or above.

**Figure 3:**
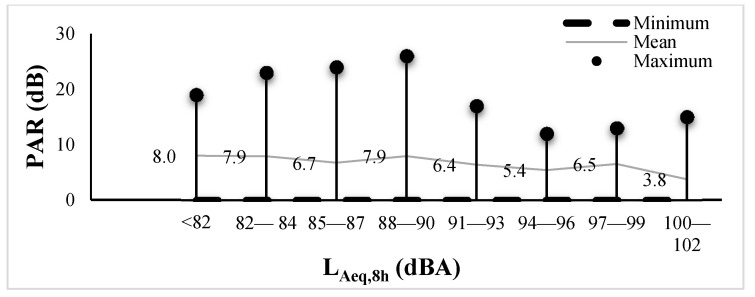
PAR ranges by L_Aeq,8h_ group of noise-exposed workers (*n* = 385). The spot line in this figure represents the mean PAR for each L_Aeq,8h_ group. There is no significant difference in the mean PARs among noise-exposed groups.

The Pearson correlation between PAR and L_Aeq,8h_ is −0.032 (*p* = 0.394). This indicates a slight negative linear relationship between PAR and L_Aeq,8h_, but it did not reach significance. Analysis of variance (ANOVA) testing shows that there was no difference in the mean PAR among L_Aeq.8h_ groups (F = 0.657, *p* = 0.709), as shown in Figure 3. Male workers achieved higher attenuation than female workers (F = 4.342, *p* = 0.038).

### 3.4. Hearing Threshold Analysis

**Figure 4:**
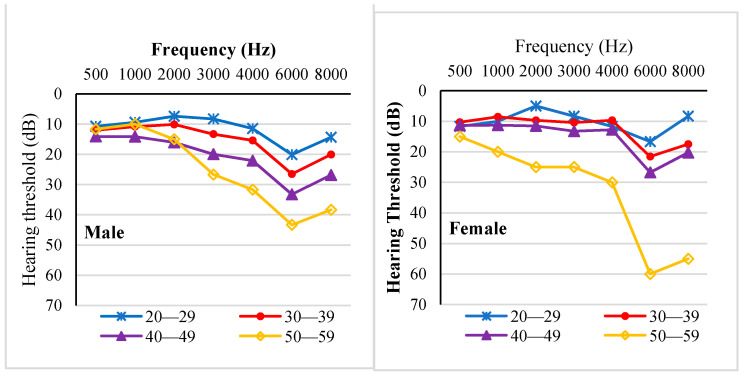
Visualized comparison of the average HTs between noise-exposed male workers (**left panel**) and female workers (**right panel**) by frequency and age group.

As age increased, so did the hearing thresholds of the noise-exposed workers from the auto parts factory in all tested frequencies (Estimate = 0.526). We observed no significant difference between left and right ears (F = 1.27, *p* = 0.259). Male workers had worse hearing than female workers (F = 107.84, *p* < 0.001), except in the 50–59 age group (Figure 4).

Noise-exposed workers’ HFHTs significantly increased as their CNE increased (t = 3.08, *p* = 0.0022). Male workers with CNE ≥ 94 dB (A)·year had significant higher average thresholds at each frequency (in the better ear) than workers with CNE < 94 dB (A)·year (F = 76.43, *p* < 0.0001). A similar observation was found for female workers (F = 11.55, *p* = 0.001) at 1000 (*p* = 0.028), 2000 (*p* = 0.030), and 6000 Hz (*p* = 0.008). Moreover, a significant difference of hearing threshold was found between male and female workers with CNE ≥ 94 dB (A)·year (F = 18.60, *p* < 0.001) at 500 (*p* = 0.041), 3000 (*p* = 0.025), and 4000 Hz (*p* = 0.002) (Table 1).

ISO-estimated median hearing thresholds (HT) were significantly better than actual thresholds of noise-exposed workers in this study (F = 170.32, *p* < 0.001) at each frequency except 4000 Hz (*p* = 0.848). Male noise-exposed workers had worse hearing threshold than non-noise-exposed male workers (F = 418.29, *p* < 0.001) at each frequency, which was similar for female workers (F = 125.86, *p* < 0.001) from 500 to 4000 Hz.

**Figure 5:**
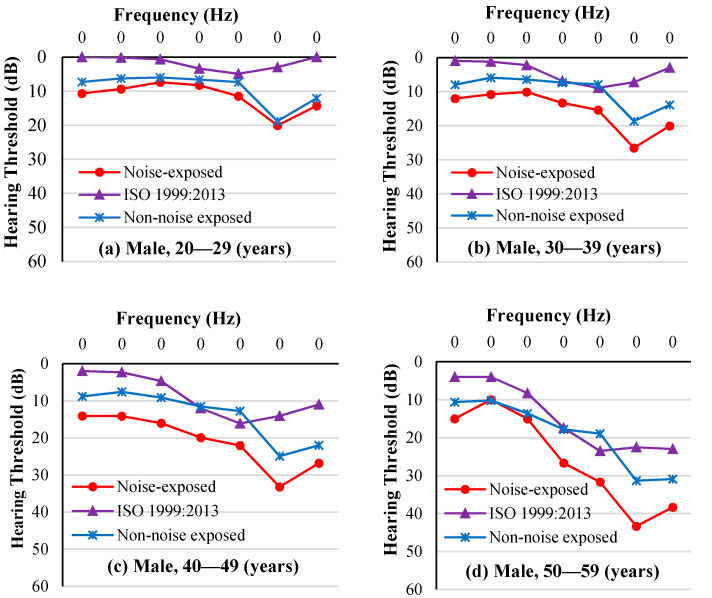
Comparison between the average hearing thresholds (HTs) of noise-exposed male workers, ISO-estimated median HTs of noise-exposed male workers, and the average HTs of non-noise-exposed male workers by frequency and age group.

**Figure 6:**
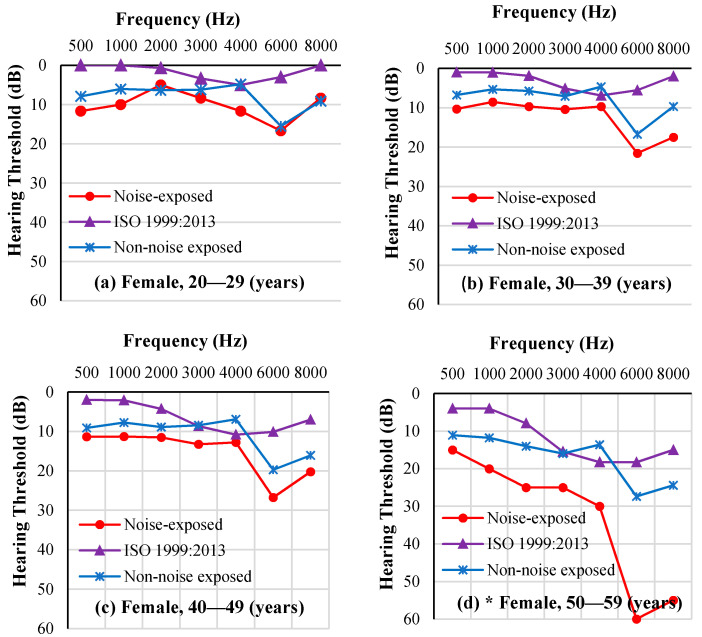
Comparison between the average HTs of noise-exposed female workers, ISO-estimated median HTs of noise-exposed female workers, and the average HTs of non-noise-exposed female workers by frequency and age group. * There was only one female worker in the 50–59 years age group.

The ISO-estimated median HT of the noise-exposed workers and the average HT of the non-noise-exposed workers, at each frequency, is presented in Figure 5 for male workers and Figure 6 for female workers. An audiometric “notch” indicating worse thresholds at 6 kHz is seen in both noise-exposed workers and non-noise-exposed workers. ISO-estimated median HTs were better than workers’ hearing thresholds at each frequency. In addition, as age increased, hearing threshold differences at each frequency between noise-exposed workers and non-noise-exposed workers increased, especially at 4, 6, and 8 kHz.

### 3.5. Evaluation of the Effectiveness of Earplug Use in the Prevention of NIHL

Sixty-five percent of the noise-exposed workers (*n* = 270) had audiometric configurations showing characteristics of a high-frequency hearing loss, a percentage significantly higher than that of non-noise-exposed study participants within gender (Table 2).

The Pearson correlation test showed that the prevalence of high-frequency hearing loss increased significantly with the increase in CNE, ACNE, and age (*p* < 0.001), while there was no difference between female and male workers (*p* = 0.995). Because the hearing thresholds used here were not adjusted by age and gender, logistic regression was conducted to evaluate their contribution to the hearing outcome. The results indicated that the prevalence of high-frequency hearing loss was associated with age (OR 1.088, 95% CI 1.047–10.131, *p* < 0.001). Males had a higher prevalence of high-frequency hearing loss than females (OR 1.720, 95% CI 1.015–2.916, *p* = 0.044). The prevalence of high-frequency hearing loss increased slightly with the CNE (OR 1.053, 95% CI 0.991–1.119, *p* = 0.097) but not significantly. The use of earplugs had no observable effect on the prevalence of high-frequency hearing loss (OR 0.964, 95% CI 0.925–1.00, *p* = 0.085).

The median HFHT at 3, 4, and 6 kHz in the better ear was 15 dB HL (mean = 19 dB HL, 95% CI 11.2 to 13.6 dB HL). An analysis with a linear model found that HFHTs significantly increased with the increase in CNE levels (t = 3.08, *p* = 0.002), females had lower hearing thresholds than males (t = 4.73, *p* < 001), and HFHTs increased by age (t = 6.00, *p* < 0.001). There was no significant association between PARs and hearing thresholds (t = −1.54, *p* = 0.125).

Both the logistic regression model and generalized linear model show consistent results indicating that high-frequency hearing loss prevalence and HFHTs increased with age and CNE levels and that female workers had better HFHTs than males. Older workers had worse hearing than younger study participants.

## 4. Discussion

The objective of this investigation was to examine earplug attenuation and its effectiveness in prevention of hearing loss among auto parts factory workers. The average L_Aeq,8h_ of 385 noise-exposed workers was 87 dB (A) with 315 workers being exposed to noise level 85 dB (A) or above. The mean PAR offered by the earplugs was 7 dB, which could theoretically protect 53.7% of the 315 noise-exposed workers by reducing in-the-ear noise exposure to less than 85 dB (A).

Many occupational and non-occupational factors can have an impact on hearing thresholds. In this investigation, we controlled for other work-related factors (ototoxicants, tenure) and examined the contribution of other factors such as age, gender, smoking, and alcohol consumption. The prevalence of high-frequency hearing loss among the noise-exposed workers was 67%, which was higher than the 33% of non-noise-exposed study participants with a hearing loss. The difference in hearing thresholds between noise-exposed workers and non-noise-exposed employees at each frequency within both male and female increased with worker age, especially at 4, 6, and 8 kHz, for both genders. This indicates that occupational noise exposure plays a significant effect on the hearing of the noise-exposed workers.

To determine whether the use of earplugs effectively protected workers from noise-induced hearing loss, two outcomes were examined: high-frequency hearing loss and HFHTs. A CNE metric that combines L_Aeq,8h_ for the duration of exposure (in work years) was used to estimate noise-exposure history. To investigate the effectiveness of HPDs based on the personal attenuation rating, the AL_Aeq,8h_ was calculated from the L_Aeq,8h_ minus the individual’s PAR, and then the ACNE was calculated from the AL_Aeq,8h_ and the exposure duration in years. If earplugs provided enough attenuation for the individual worker, both the prevalence of high-frequency hearing loss and HFHT at a level of CNE should be higher (worse hearing) than that at the same level of ACNE. To test this hypothesis, the difference between CNE and ACNE at the same level was used as an indicator of earplug effectiveness.

Both the generalized linear model and logistic regression model showed that earplug attenuation had no significant effect on the hearing of participants. These findings are in agreement with the evidence from previous studies that the use of HPDs in the workplace is often inconsistent and not fully effective [7,9,18,19,20,21,22]. Specifically, these findings are consistent with Groenewold’s [5] report which showed self-reported use of hearing protection devices at work was not significantly associated with the odds of high-frequency threshold shifts among 19,911 noise-exposed U.S. workers. The results from this study not only confirmed that the real-world attenuation received was much lower than the labeled attenuation values obtained in the controlled laboratory testing, but also indicated that simply requiring earplugs to be worn for several years had no detectable effect on the prevention of hearing loss at the auto parts factory.

There are many possible explanations for workers not achieving the desired attenuation. Poor fitting techniques (e.g., not effectively straightening out the ear canal before inserting an earplug) were observed by the research staff. When the researcher attached the dual-element microphone array to the probed test earplugs, the researcher could often feel that the earplugs did not seal these workers’ ear canals. A wide range of PAR values from 0 to 25 dB was observed among workers who improperly fit their earplugs. More than half of the workers achieved 5 dB or lower attenuation. These results, however, must be interpreted with caution. Workers might have worn earplugs differently or even fit them better than they normally do because they realized that they were being tested. Therefore, it is conceivable that the amount of attenuation the workers received on a typical workday is different from what was obtained in this study. Still, they are a robust illustration of the challenges in achieving the desired earplug attenuation.

Some workers were observed not wearing earplugs all the time during their full shift even though they were required to do so. Some workers took their earplugs off when they needed to communicate. Similar observations were made by Nélisse [23], who tested various types of earmuffs and one type of molded earplug in 24 workers from eight different companies. During shifts, workers had their earplugs regularly removed and poorly inserted. The PAR data showed considerable fluctuations over an entire work shift. Neitzel and Seixas [24] concluded that HPDs provided construction workers only 3 dB of effective noise attenuation and reduced hazardous noise exposures to safe levels only about 20% of the time. In this study, we tested PARs once with the earplug before workers’ audiological testing.

In addition, only one type of premolded earplug was offered to the workers in the 6-year period that preceded this study. A previous survey conducted by the JSCDC for 503 noise-exposed workers at nine manufacturing facilities in China revealed that 10% of the workers were unable to achieve target attenuation from the assigned HPD after individual training. For those who needed and were provided a different product or size of HPD, 70% were able to achieve the target attenuation [18]. Similar results were found in the earplug fit-testing study conducted by Murphy et al. in 2012. Murphy reported that among 75 workers, 15 workers did not achieve a target PAR with the protectors they usually wore after individual training. However, these 15 workers who were retested with a different earplug all were able to achieve the target PAR [7].

Finally, the group training method may have contributed to the lack of effectiveness of earplugs observed in this study. The surveyed auto parts factory had a well-organized occupational health and safety program, in which workers received a group pre-employment training and annual refresher training followed by a written exam. However, multiple studies of the training methods have shown that they failed to teach how to properly fit their earplugs [9,19,20,21]. Individual training along with HPD fit testing has been shown to teach poorly performing subjects to insert earplugs properly and achieve sufficient attenuation [7,9,21,22]. To improve earplug fitting skill and to train workers to wear them correctly, individual fit-testing was recommended to the surveyed auto parts factory. The surveyed factory was also encouraged to offer workers a wide selection of HPDs to choose from.

In summary, despite being required for almost a decade, as worn by this group of workers, earplugs generally did not provide the needed attenuation and consequently showed no significant protective effect on the hearing of the participants. Similar findings have been reported earlier [3,4,5]. Even though the surveyed factory had a well-organized occupational health and safety program, we could not obtain any evidence of its effectiveness. Improper fitting and inconsistent use of earplugs are causally related to the workers’ overexposure to hazardous noise. This is probably the leading cause of the high hearing loss prevalence observed in the surveyed factory. Recommendations included supervision of workers on consistent use of earplugs and modification of workplace policy for the occupational health and safety program.

Additional findings from this study provide insight that could prove valuable in increasing the effectiveness of hearing protection programs. First, no PAR increment was detected with increasing L_Aeq,8h_. This finding was different from the findings of Chen et al. [10]. They reported that workers in areas with a higher noise exposure level might be more bothered by noise and consequently be more conscious about the use of HPDs. In this study, the workers exposed to noise at higher levels did not obtain higher PARs. More studies are needed on this issue.

Second, this study found that the ISO-estimated median HTs were significantly better than the real-world thresholds of noise-exposed workers in this study. Differences in the population characteristics and noise exposure estimations might explain this finding. The study was able to conduct noise measurements for all participants and incorporate kurtosis in the calculation of a cumulative noise estimate. Kurtosis has been reported to play an important role in evaluating NIHL [13,14] and earplug attenuation [25]. Studies showed that for a fixed noise energy level and spectrum, NIHL increased as the kurtosis of the noise exposure increased [13,26,27]. The kurtosis of Gaussian noise is 3, while the mean kurtosis for the 385 noise-exposed workers in this study was 19.8 with an SD of 12.6, indicating that most workers are exposed to non-Gaussian noise. The prevalence of NIHL adjusted by age and gender among the workers exposed to 94 ≤ CNE ≤ 100 dB (A) year was 41%, which was higher than in the Gaussian noise group (11.10%) at 95 ≤ CNE < 100 dB (A)·year in Xie’s study [14]. This result demonstrated the importance of kurtosis in evaluating NIHL and the noise attenuation performance of HPDs.

Third, the Pearson correlation test showed no difference in the hearing outcomes between female and male noise-exposed workers (*p* = 0.995). In this study, the average age of male noise-exposed workers was 5 years younger than female noise-exposed workers. Examining age and gender through a logistic regression model and in a generalized linear model, age was shown to have a significant effect on the noise-exposed workers’ hearing. This finding is consistent with the reports from Groenewold et al. [5] and Lee et al. [28]. It is unclear whether this results from intrinsic biological differences or extrinsic lifestyle differences.

### Limitations of the Study

The case-control design and having only one instance of each of the measurements (noise, hearing thresholds, and PAR) are the important limitations of this study. Of all information collected, noise exposures were the most robust of the studied variables. We were unable to use historical audiometric data from the noise-exposed workers, as companies are only required to do a periodic pass/fail hearing screening. In addition, we only conducted fit testing once. A single PAR result cannot sufficiently represent the attenuation obtained throughout the years, perhaps not even in a day. Studies with repeated measurements could provide more robust evidence on the real-world attenuation provided by the HPDs. Although the workers were exposed to eight different types of work noise, the generality of the findings in this study may be limited by the fact that the data came from a single factory. Similar studies in different industries will be helpful to further evaluate the actual protection capability of HPDs in the real world.

## 5. Conclusions

Despite these limitations, we observed an association between noise exposure and the hearing of this population of relatively young workers and found no evidence of any significant contribution from the existing hearing protection program. In current practice, the use of earplugs had limited effectiveness in the prevention of hearing loss at the auto parts factory. In addition, a single choice of HPDs for the workers could be a barrier to an effective hearing loss prevention program. Our study suggests that several programmatic elements can impact the success of hearing loss prevention initiatives. Periodic hearing tests, earplug fit testing, expanding the offer of different types of hearing protection, and employee education about the importance of protecting their hearing were recommended to the surveyed factory’s occupational health and safety program.

## Figures and Tables

**Table 1:** Workers’ average hearing thresholds at each frequency in better ear by gender, frequency, and cumulative noise exposure (CNE).

Gender	CNE dB (A)·Year	Hearing Thresholds (dBA)						
		500 Hz	1000 Hz	2000 Hz	3000 Hz	4000 Hz	6000 Hz	8000 Hz
Female	<94	11.2	8.3	8.8	12.4	11.0	22.1	18.1
	≥94	10.9	10.8 *	11.3 *	11.9	11.8	25.5 *	19.4
Male	<94	10.6	9.4	8.1	10.1	12.9	21.1	15.2
	≥94	13.0 *^,†^	12.3 *	12.6 *	15.8 *^,^^†^	18.1 *^,^^†^	29.6 *	23.2 *

* The average thresholds in better ear of noise-exposed workers with CNE ≥ 94 dB (A)·year were significantly higher than workers with CNE < 94 dB (A)·year within female and male workers (*p* < 0.05). ^†^ The average thresholds in better ear of noise-exposed male workers with CNE ≥ 94 dB (A)·year were significantly higher than those of female workers with CNE ≥ 94 dB (A)·year (*p* < 0.05).

**Table 2:** Prevalence of audiograms with a high-frequency notch configuration by group, age, and gender of the study participants.

Age (Years)	Male		Female	
	Noise-Exposed Workers	Non-Noise-Exposed Workers	Noise-Exposed Workers	Non-Noise-Exposed Workers
22–29	46%	18%	0%	15%
30–39	66%	32%	54%	15%
40–49	83%	50%	76%	31%
50–59	67%	75%	100%	59%
Total	65%	44%	65%	24%

## Data Availability

The data that support the findings of this study are available from the first author [W.G.] upon reasonable request.

## References

[1] Blackwell D.L., Lucas J.W., Clarke T.C. (2014). Summary Health Statistics for U.S. Adults: National Health Interview Survey, 2012. Vital and Health Statistics. Series 10, Data from the National Health Survey.

[2] China MoHotPsRo (1999). National Health of the People’s Republic of China Chinese national standard. Standards for Hearing Protection for Workers in Industrial Enterprises.

[3] Heyer N., Morata T.C., Pinkerton L.E., Brueck S.E., Stancescu D., Panaccio M.P., Kim H., Sinclair J.S., Waters M.A., Estill C.F. (2011). Use of historical data and a novel metric in the evaluation of the effectiveness of hearing conservation program components. Occup. Environ. Med..

[4] Tikka C., Verbeek J.H., Kateman E., Morata T.C., Dreschler W.A., Ferrite S. (2017). Interventions to prevent occupational noise-induced hearing loss. Cochrane Database Syst. Rev..

[5] Groenewold M.R., Masterson E.A., Themann C.L., Davis R.R. (2014). Do hearing protectors protect hearing?. Am. J. Ind. Med..

[6] (SAWS), State Administration of Work Safety of the People’s Republic of China (2018). Norm for Employers on Administration of Personal Protective Equipment.

[7] Murphy W.J., Themann C.L., Murata T.K. (2016). Hearing protector fit testing with off-shore oil-rig inspectors in Louisiana and Texas. Int. J. Audiol..

[8] Berger E.H., Franks J.R., Behar A., Casali J.G., Dixon-Ernst C., Kieper R.W., Merry C.J., Mozo B.T., Nixon C.W., Ohlin D. (1998). Development of a new standard laboratory protocol for estimating the field attenuation of hearing protection devices. Part III. The validity of using subject-fit data. J. Acoust. Soc. Am..

[9] Gong W., Liu X., Liu Y., Li L. (2019). Evaluating the effect of training along with fit testing on foam earplug users in four factories in China. Int. J. Audiol..

[10] Chen Y., Zhang M., Qiu W., Sun X., Wang X., Dong Y., Chen Z., Hu W. (2019). Prevalence and determinants of noise—induced hearing loss among workers in the automotive industry in China: A pilot study. J. Occup. Health.

[11] NIOSH Criteria for a Recommended Standard: Occupational Noise Exposure: Revised Criteria, 1998. U.S. Department of Health and Human Services, Public Health Service, Centers for Disease Control and Prevention, National Institute for Occupational Safety and Health, DHHS (NIOSH) Publication No. 98–126, 1998. https://www.cdc.gov/niosh/docs/98-126/pdfs/98-126.pdf.

[12] Fuente A., Qiu W., Zhang M., Xie H., Kardous C.A., Campo P., Morata T.C. (2018). Use of the kurtosis statistic in an evaluation of the effects of noise and solvent exposures on the hearing thresholds of workers: An exploratory study. J. Acoust. Soc. Am..

[13] Zhang M., Xie H., Zhou J., Sun X., Hu W., Zou H., Zhou L., Li J., Zhang M., Kardous C.A. (2021). New Metrics Needed in the Evaluation of Hearing Hazard Associated with Industrial Noise Exposure. Ear Hear..

[14] Xie H.W., Qiu W., Heyer N.J., Zhang M.B., Zhang P., Zhao Y.M., Hamernik R.P. (2016). The Use of the Kurtosis-Adjusted Cumulative Noise Exposure Metric in Evaluating the Hearing Loss Risk for Complex Noise. Ear Hear..

[15] Earshen J.J., Berger E.H., Ward W.D., Morrill J.C., Royster L.H. (1986). Chapter 3: Sound Measurement: Instrumentation and Noise Descriptors. Noise and Hearing Conservation Manual.

[16] Hoffman H.J., Dobie R.A., Ko C.-W., Themann C.L., Murphy W.J. (2010). Americans Hear as Well or Better Today Compared with 40 Years Ago: Hearing Threshold Levels in the Unscreened Adult Population of the United States, 1959–1962 and 1999–2004. Ear Hear..

[17] Berger E.H., Voix J., Hager L.D. (2008). Methods of fit testing hearing protectors, with representative field test data. Hearing Loss: 9th International Congress on Noise as a Public Health Problem (ICBEN).

[18] Gong W., Liu X., Liu Y., Li L. (2021). Verifying earplug attenuation and evaluating the effectiveness of one-on-one training along with earplug fit-testing at nine facilities in China. Am. J. Ind. Med..

[19] Murphy W.J., Stephenson M.R., Byrne D.C., Witt B., Duran J. (2011). Effects of training on hearing protector attenuation. Noise Heal..

[20] Kim J.W., Yang S., Chung I., Lee M.-Y. (2019). The effect of earplug training on noise protection. Ann. Occup. Environ. Med..

[21] Liu Y., Gong W., Liu X., Li L. (2019). Evaluating the Effect of Training Along with Fit Testing on Premolded Earplug Users in a Chinese Petrochemical Plant. Ear Hear..

[22] Chiu C.C., Wan T.J. (2020). Individual Fit Testing of Hearing-Protection Devices Based on Microphones in Real Ears among Workers in Industries with High-Noise-Level Manufacturing. Int. J. Environ. Res. Public Health.

[23] Nélisse H., Gaudreau M.-A., Boutin J., Voix J., Laville F. (2011). Measurement of Hearing Protection Devices Performance in the Workplace during Full-Shift Working Operations. Ann. Occup. Hyg..

[24] Neitzel R., Seixas N. (2005). The Effectiveness of Hearing Protection Among Construction Workers. J. Occup. Environ. Hyg..

[25] Murphy W. The Effect of Hearing Protection on Kurtosis. Proceedings of the INTER-NOISE 2019 MADRID.

[26] Qiu W., Zhang M., Hamernik R. (2015). Role of the kurtosis metric in evaluating hearing trauma from complex noise exposures—From animal experiments to human applications. J. Acoust. Soc. Am..

[27] Davis R.I., Qiu W., Hamernik R.P. (2009). Role of the Kurtosis Statistic in Evaluating Complex Noise Exposures for the Protection of Hearing. Ear Hear..

[28] Lee J.S., Choi H.G., Jang J.H., Sim S., Hong S.K., Lee H.-J., Park B., Kim H.-J. (2015). Analysis of Predisposing Factors for Hearing Loss in Adults. J. Korean Med. Sci..

